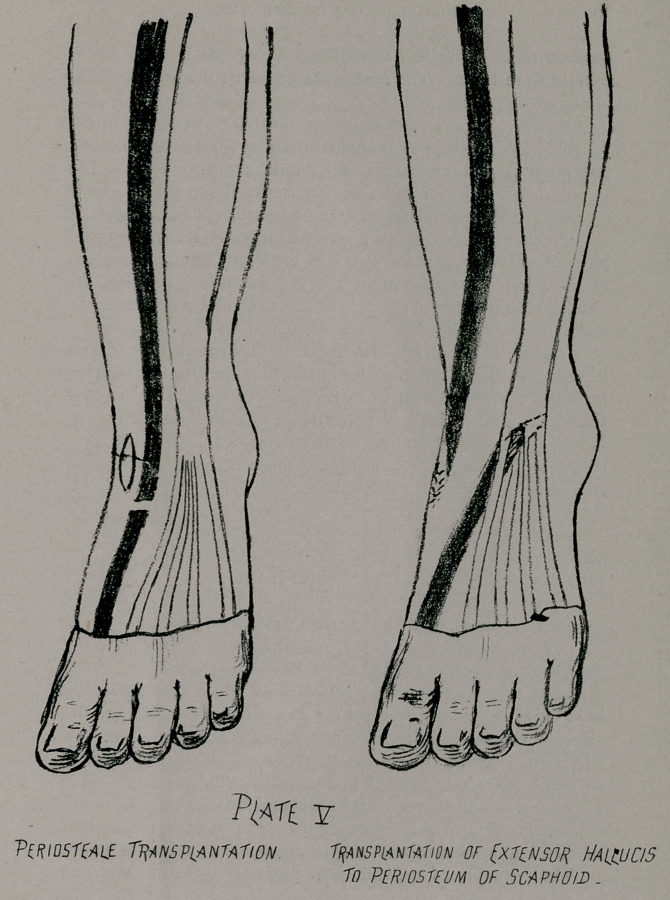# Tenotomy

**Published:** 1910-05

**Authors:** Theodore Toepel

**Affiliations:** Atlanta, Ga.


### TENOTOMY.

THEODORE TOEPEL, M. D., ATLANTA, GA.

Two principal methods of tenotomy are in general use, the subcutaneous and the open operation.

The subcutaneous was first performed by Stromeyer in 1831 when he severed the Tendo-Achillis of a nineteen year old patient. This method is still employed a great deal, especially in lengthening of tendons, and the open method is used whenever it is considered safer to expose the tendons and avoid injury to the adjacent structures.

The term tenotomy embraces lengthening or shortening of tendons and tendon transplanation.

According to Wilson, tendon splicing was first suggested by Rhoades of Philadelphia, was first performed by Anderson, and was first described by Keen.

The dividing of a tendon is done by various methods, the one I prefer is to make a longitudinal section, then the upper and lower portions of the tendon are divided by a cross-cut, from the longitudinal incision outward, thus making a section

PLATE I

LENGTHENING OF TENDO ACHILLIS

BY DIVIDING AND SLIPPING THE TWO

HALVES OVER EACH OTHER

SUTURE AFTER THE OPERATION

as illustration shows (Plate I.) Since this method was first performed by Anderson, it is known as the Anderson method.

Immediately following a well performed operation of lengthening a tendon by either the open or subcutaneous method, the process of repair begins by the effusion of a fluid, or semi-fluid lymph from the tendon endings and tendon sheath. Usually very little blood is effused into the space, if much blood has been effused, it retards the formation of the proper exudate. This exudate takes no part in the reparative process and usually ceases in twenty-four hours. From three to seven days after this the reparative cells make their appearance and fill the entire space between the divided entremities. It is very interesting to observe that this reparative exudate is always sufficient to fill the space between the severed ends, which in some instances is very great. Dr. Willard showed me a case where the ends were separated three inches and firm union had occurred.

TECHNIC. In performing subcutaneous tenotomy much of the success depends on the technic. It is well to have tenotomes of various shapes and cutting surfaces. (Plate II.) Anesthesia is not absolutely essential, but is usually employed to overcome movements on the part of the patient which might interfere with the operation. It is needless to go into details as to the preparation of the part for the operation before a body of intelligent physicians, except to say that the aseptic preparation of the part must be very carefully carried out, particularly where the open operation is performed, this caution also applies to the surgeon. The tenotome is carried flatwise beneath and close to the under surface of the tendon, the cutting edge is turned against it and the division accomplished by a slight rocking motion. The puncture is dressed inthe usual way, wet bichloride compress, gauze and cotton and then the parts are placed in a slightly overcorrected position and retained in position by a plaster dressing. The wound need not be inspected for ten days unless pain or elevation of temperature calls attention to the part.

*After Treatment* requires as much if not more attention to detail than the operation itself, and on this depends much of the success. At the expiration of ten days the parts should be inspected and if in good condition it should be placed in an

PLATE II. TENOTOMES

adjustable retentive apparatus. At least once a day the parts should be massaged and manipulated, particularly over the muscles and tendons which were formerly contracted. This is very important and tends to free and strengthen the tendons. The retentive apparatus must be worn day and night so as to prevent any tendency to relapse. Electricity should be applied to the whole part to improve the nutrition of the skin and deeper structures, but it should be more particularly applied to paralyzed muscles.

*Tendon Shortening.* Tendons may be shortened by an oblique division and an overlapping of the ends or they may be folded upon themselves, or they may be ruffled. In shortening a tendon by the first method, as the Tendon Achillis, the tendon must be exposed by a longitudinal incision and divided in the direction of the fibres by a central incision which is cut out at each end. The cut surfaces are then slid past each other and the deformed part is placed in such a manner that the overlapping may be accomplished. Very fine sutures are inserted to hold the cut surfaces in position, the sheath is held together with a second row of sutures, and the skin is held together with a third row of sutures. The usual dressing is then applied and the parts are held in position by a plaster bandage.

*Transplantation of Tendons.* There are three methods of transplanting tendons. The first consists in dividing the tendon of a sound muscle and inserting it into a slit in the paralyzed muscle; the distal extremity of the divided tendon is again attached in the same manner in an upward direction into a tendon of another sound muscle. In this manner the tendon of the extensor proprius hallucis may be attached to the tibialis anticus and the distal extremity may be attached to the extensor longus digitorum, (Plate III.) The second method consists in taking a slip from a sound tendon and inserting it into a slit in a paralyzed tendon, in this fashion a slip from the extensor proprius hallucis may be attached into a slit of the tibialis anticus, (Plate IV.) The third method consists in dividing a sound tendon and attaching it near the insertion of the paralyzed tendon. The distal extremity is then attached to another sound tendon. For example, the sound tendon of the extensor proprius hallucis may

PLATE III

PARALIZED TIBIALIS ANTICUS

COMPLETE TRANSPLANTATION OF EXTENSOR HALLUCIS TO TIBIALIS ANTICUS

be attached to the periosteum of the scaphoid bone, the distal extremity of the cut tendon then being attached to the extensor longus digitorum (Plate V.) The greatest care must be observed in the technic of this operation to have it thoroughly aseptic. The method that I have found the simplest and the best of transplanting tendons is to carry the sound tendon into a slit which has been made in the paralyzed tendon, securing it in place with interrupted sutures. Where tendons are carried for some distance for purpose of transplanting; tunnels must be made for them with a blunt instrument beneath the subcutaneous tissue. The advantage of attaching tendons at a new point in the periosteum is that the operator has a free selection of the point of insertion for the transplanted tendon. Where the tendon is too short to reach the periosteal insertion, it may be attached by silk strands, which afterwards become enclosed in a deposit of fibrous tissue, layer upon layer, so that it, after a time, becomes a tendinous band in the centre of which is the silk thread. The after treatment is the same as described heretofore.

It is desirable to fix the parts preceding the operation for six to eight weeks in the expectant ultimate position, this preparation will enable a more accurate approximation of the tendons with no danger to tearing out of sutures.

Satisfactory results of transplanted tendons depend to a great extent upon the following enumerated technical points: I. Make correct diagnosis of paralyzed muscles and make your plan for operation accordingly, and observe strict technic of transplantation minutely and faultlessly with strict asepsis. II. Fix parts carefully in a slightly over-corrected position and apply plaster of Paris retentive dressing carefully. III. Be careful in manipulation of the parts in after-treatment and the accurate re-application of the retentive apparatus to prevent tearing of the approximated tendons.

*Case I.*—Anterior poleomyelitis, right leg involved. Child, C. C., age 9, was first seen in the fall of 1907. The child at the age of three was suddenly seized by a vomiting spell while at walk. She was unable to rise and had to be carried home, where for several days she had a high fever. Osteopathy was used for three years. Examination revealed that the right leg

PLATE IV.

PARTIAL TRANSPLANTATION OF EXTENSOR HALLUCIS TO TIBIALIS ANTICUS

was very much atrophied, comparative measurements of thighs showed a difference of one and one-half inches and at the calf one inch. There was a shortening of the leg of one-half inch, the heel did not touch the ground on account of contracted Tendon Achillis, (Talipes Equinus).

*Treatment.*—Massage, active and resistive exercises with electricity were given as indicated by progress of treatment. Outdoor living and a nutritious diet were prescribed and the patient by this treatment gradually improved. The difference of comparative measurements of thighs was reduced to one-half inch and that of the calves to one-quarter inch. The Anderson tenotomy of the Tendon Achillis was done and the child is now able to bring the heel down and to walk more naturally.

*Case. II.*—Infantile paralysis (Hemiplegia) involving the whole left side. Child S. S., age 12, first saw her in May, 1909. Examination showed marked assymetry of the face, head was bent to the right. The comparative measurements varied from 0.3 of an inch to 2 inches. Spine showed in the dorsal region lateral deviation of 0.8 of an inch. Left foot was in a talipes valgus position.. Flexors and extensors of hand and forearm were involved, the extensors being more affected, so that the hand was in a habitual attitude of palmar flexion of a right angle with adduction of thumb. Contractile power of left hand was two pounds and that of right hand was thirty-four pounds. This patient had never been treated.

*Treatment.*—Outdoor active exercises, nutritious food, cold sponge bath with vigorous rub down, localized resistive exercises to improve the undersized muscles and electricity were ordered. Special attention was given to the hand and foot. A special ankle and foot brace was worn and corrective exercises given with good results. The hand was carried in a over-corrected position, thumb abducted in a light removable plaster cast for two months in order to relax the flexor tendons and prepare for shortening of extensor tendons by operation. The tendons of the extensor communis digiti were shortened and the adductor pollicis transversus and adductor pollicis obliquos were divided in order to move the thumb out of the way of the index finger. The result of the operation has been very satisfactory as far as

PLATE V

PERIOSTEALE TRANSPLANTATION. TRANSPLANTATION OF EXTENSOR HALLUCIS TO PERIOSTEUM OF SCAPHOID.

appearance is concerned, three times a day passive and active exercises are taken. The contractile power of the left hand has increased eight pounds.

*Case III.*—Anterior poleomyelitis, involving left leg and foot. A boy six years old was brought to me by a masseur who had given the patient massage and exercise for two years. The treatment given up to this time had restored the action of the muscles and had stimulated growth of muscular fibres.

Examination showed that the patient was in a fairly good physical condition, but the boy was unable to flex his foot due to the paralyzed condition of the tibialis anticus muscles, the Tendo Achillis had not contracted, due to the persistent continuous exercise. I suggested operation, which was accepted.

*Treatment.*—Applied plaster cast to the parts in a dorsal flexed position and kept the foot in this condition for two months, taking it out of the cast three times a day for the purpose of massage, active exercise and electricity. I then performed complete tendon transplantation of the extensor hallucis and proceeded with the after treatment as outlined heretofore with good results. The boy now walks naturally.

## Figures and Tables

**PLATE I f1:**
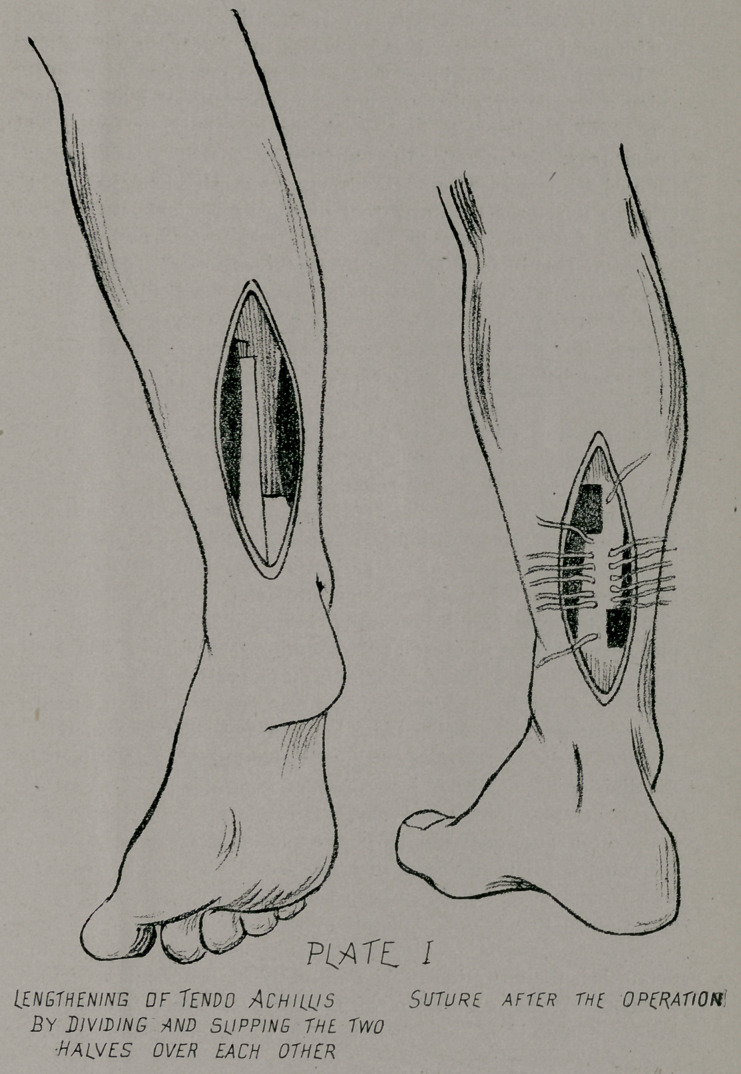


**PLATE II. f2:**
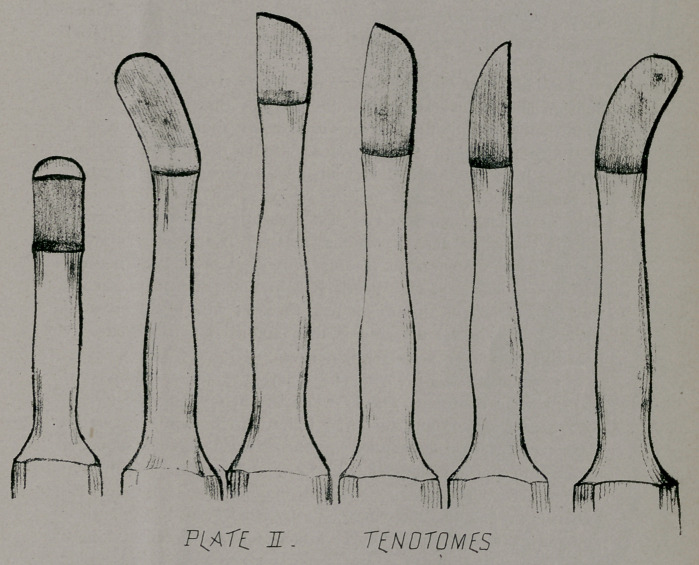


**PLATE III f3:**
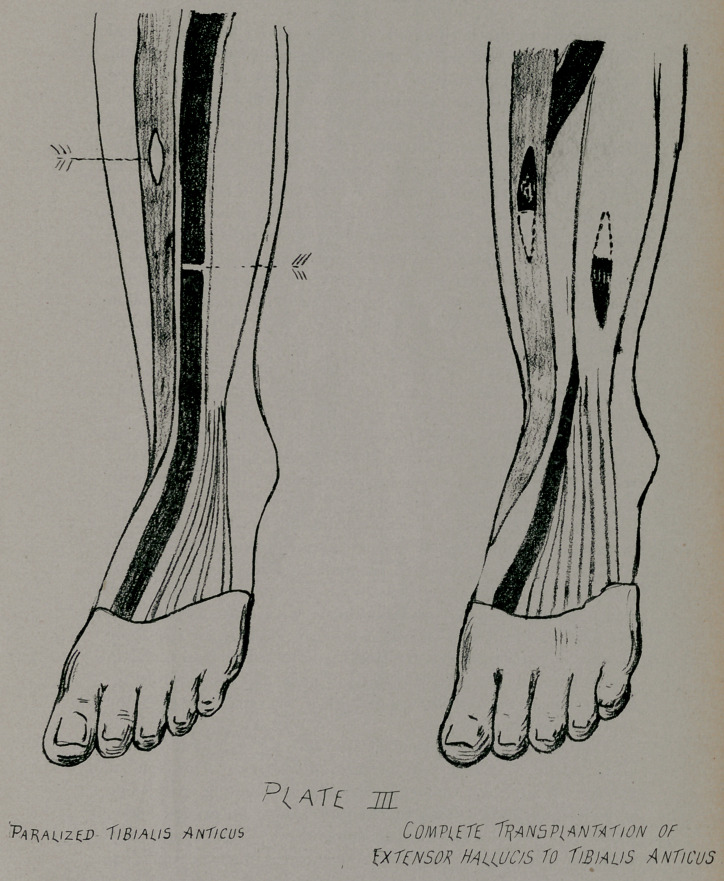


**PLATE IV. f4:**
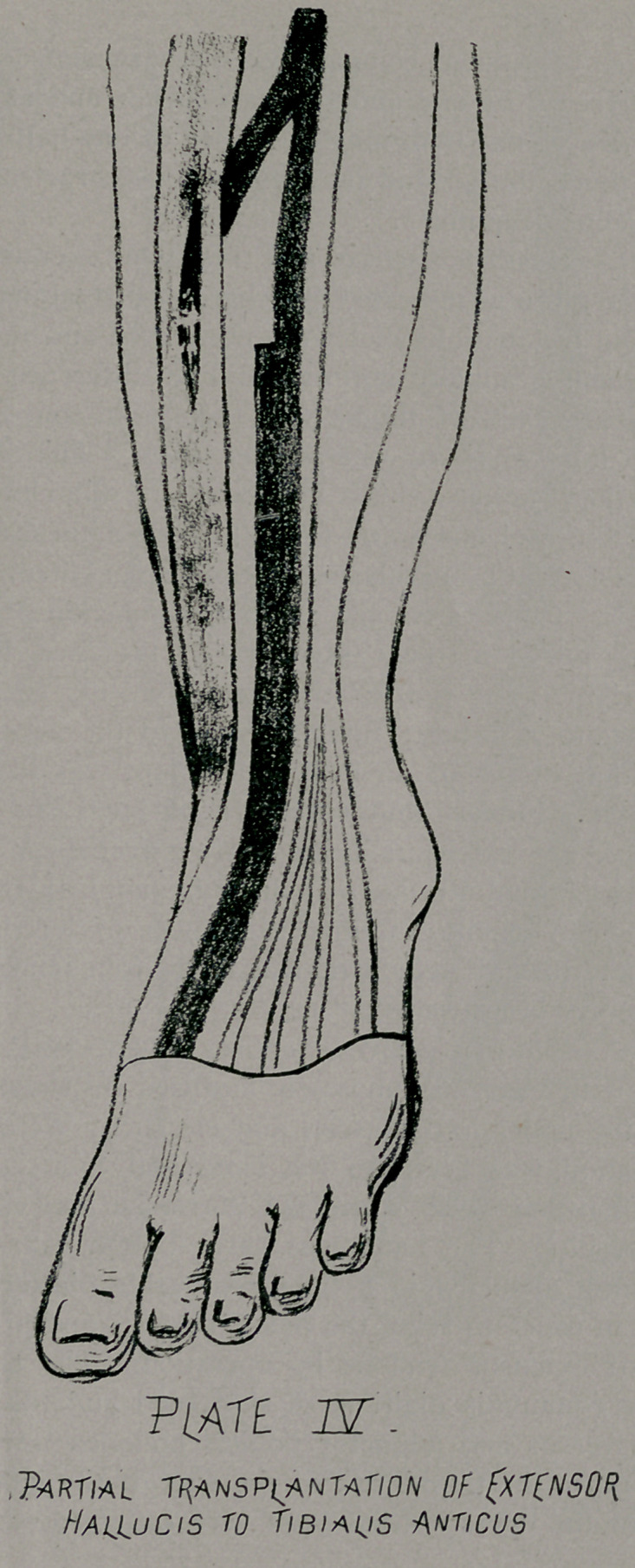


**PLATE V f5:**